# Society Meetings

**Published:** 1908-05

**Authors:** 


### SOCIETY MEETINGS.

The Medical Society of the County of Erie held its regular quarterly meeting Monday, April 20, 1908, under the presidency of Dr. Edward Clark, with the following program: morning session, medical clinic by Charles Cary at the Buffalo General Hospital, at 10 o'clock; surgical clinic by Roswell Park at the same place, 11 o'clock; afternoon session, business meeting, 4 o'clock, at the Buffalo library building; evening session, 8.30 o'clock, at same place: Five minute talks: Thomas B. Carpenter, Albumin; Eugene A. Smith, Intussusception; George F. Cott, Probability of Intracranial Complications Following Middle Ear Infection; H. C. Rooth, Radical Treatment of Incipient Hip-joint Disease. Report of Case; A. E. Woehnert, Casts; J. E. King, Influence of Relaxed Uterosacrals on Causation of Symptoms in Retroversion; William C. Krauss, Diagnosis of Spinal Cord Tumors—report of three cases seen within a period of ten days.

---

The following medical meetings will be held at Chicago, during the first week in June, 1908:

American Medical Association, June 2 to 5.

American Academy of Medicine, May 30, and June 1.

National Confederation of Medical Examining and Licensing Boards, June 1.

National Association of U. S. Pension Examining Surgeons, Palmer House, June 1.

Association of American Teachers of the Diseases of Children, Great Northern, June 1.

American Medical Editors' Association, May 30, June 1.

American Proctologic Society, June 1 and 2.

American Gastro-Enterological Association, June 1, and 2.

Secretaries and Editors of State Journals, June 1.

---

The American Therapeutic Society will hold its ninth annual meeting at the Bellevue-Stratford Hotel, Philadelphia, May 7 to 9, 1908.

---

The Mississippi Valley Medical Association will hold its thirty-fourth annual meeting at Louisville, October 13 to 15, 1908, under the presidency of Dr. Arthur R. Elliott, of Chicago. Dr. Henry Enos Tuley is the secretary and Dr. Louis Frank is the chairman of the committee of arrangements.

---

The Buffalo Academy of Medicine held meetings during April as follows:

*Section on Surgery.*—Tuesday evening, April 7. Program: Acute traumatic and chronic synovitis of the knee, Robert W. Lovett, Boston.

*Section on Medicine.*—Tuesday evening, April 14. Program: (*a*) Apoplexy, its causes and treatment, John D. Bonnar; (*b*) Psychotherapy, Julius Ullman. The discussion was opened by James W. Putnam.

*Section on Pathology.*—Tuesday evening, April 21. Program: (*a*) Presentation of specimens of variations seen in livers hardened in situ, James A. Gibson; (*b*) Transplantation of suprarenal gland, F. C. Busch.

The Rochester Academy of Medicine held meetings during April as follows:

*Section I. General Medicine.*—Wednesday evening, April 1, 1908. Program: Symposium on pneumonia. Bacterial origin and factors entering into causation, John R. Williams; The blood, Charles O. Boswell; Treatment, J. R. Culkin; Treatment (specific), nuclein and serum therapy, C. E. Darrow.

*Section II. Surgery.*—Wednesday evening, April 8. Program: Report of a case of retention of urine complicating pregnancy, Floyd S. Winslow; report of two cases of tubercular peritonitis, Floyd S. Winslow, (by invitation); Report of a case of skin grafting, Milton Chapman, (by invitation).

*Section III.* Obstetrics, Gynecology, and Pediatrics. Wednesday evening, April 15. Program: Case reports, Charles R. Barber; obstetric hemorrhages: causative factors and methods of control with case reports, William M. Brown.

*Section IV. Public Health.*—Wednesday evening, April 22, Program: Climatology. Rochester has a superior climate! Illustrated talk by Professor Fairchild. (By invitation.)

---

The Buffalo General Hospital Alumni Association held its annual meeting at the Lenox Hotel, Friday evening, April 24, 1908. Dr. E. L. Shurly, of Detroit, was announced to deliver an address on "The Hospital Interne;" Dr. H. R. Hopkins to give a talk on "Quinby-Eddy Diabolism;" Drs. William C. Phelps, P. W. Van Peyma, DeLancey Rochester and John Parmenter were expected to respond to the toasts.

